# The Impact of Diet on the Involvement of Non-Coding RNAs, Extracellular Vesicles, and Gut Microbiome-Virome in Colorectal Cancer Initiation and Progression

**DOI:** 10.3389/fonc.2020.583372

**Published:** 2020-12-14

**Authors:** Bene A. Ekine-Afolabi, Anoka A. Njan, Solomon O. Rotimi, Anu R. I., Attia M. Elbehi, Elizabeth Cash, Ademola Adeyeye

**Affiliations:** ^1^ ZEAB Therapeutic, London, United Kingdom; ^2^ Cancer Biology and Therapeutics, High Impact Cancer Research Postgraduate Certificate Program, Harvard Medical School, Boston, MA, United States; ^3^ Department of Pharmacology and Therapeutics, Faculty of Basic Medical Sciences, College of Health Sciences, University of Ilorin, Ilorin, Nigeria; ^4^ Department of Biochemistry, Covenant University, Ota, Nigeria; ^5^ Department of Clinical Biochemistry, MVR Cancer Centre and Research Institute, Calicut, India; ^6^ School of Care and Health Sciences, University of South Wales, Cardif, United Kingdom; ^7^ Department of Otolaryngology-Head and Neck Surgery and Communicative Disorders, University of Louisville School of Medicine, Louisville, KY, United States; ^8^ Department of Surgery, University of Ilorin Teaching Hospital, Ilorin, Nigeria

**Keywords:** diet, ncRNAs, extracellular vesicles, cancer prevention, colorectal cancer, microbiome, GSH, MGMT

## Abstract

Cancer is the major cause of morbidity and mortality in the world today. The third most common cancer and which is most diet related is colorectal cancer (CRC). Although there is complexity and limited understanding in the link between diet and CRC, the advancement in research methods have demonstrated the involvement of non-coding RNAs (ncRNAs) as key regulators of gene expression. MicroRNAs (miRNAs) which are a class of ncRNAs are key players in cancer related pathways in the context of dietary modulation. The involvement of ncRNA in cancer progression has recently been clarified throughout the last decade. ncRNAs are involved in biological processes relating to tumor onset and progression. The advances in research have given insights into cell to cell communication, by highlighting the pivotal involvement of extracellular vesicle (EV) associated-ncRNAs in tumorigenesis. The abundance and stability of EV associated ncRNAs act as a new diagnostic and therapeutic target for cancer. The understanding of the deranging of these molecules in cancer can give access to modulating the expression of the ncRNAs, thereby influencing the cancer phenotype. Food derived exosomes/vesicles (FDE) are gaining interest in the implication of exosomes in cell-cell communication with little or no understanding to date on the role FDE plays. There are resident microbiota in the colon; to which the imbalance in the normal intestinal occurrence leads to chronic inflammation and the production of carcinogenic metabolites that lead to neoplasm. Limited studies have shown the implication of various types of microbiome in CRC incidence, without particular emphasis on fungi and protozoa. This review discusses important dietary factors in relation to the expression of EV-associated ncRNAs in CRC, the impact of diet on the colon ecosystem with particular emphasis on molecular mechanisms of interactions in the ecosystem, the influence of homeostasis regulators such as glutathione, and its conjugating enzyme-glutathione S-transferase (GST) polymorphism on intestinal ecosystem, oxidative stress response, and its relationship to DNA adduct fighting enzyme-0-6-methylguanine-DNA methyltransferase. The understanding of the molecular mechanisms and interaction in the intestinal ecosystem will inform on the diagnostic, preventive and prognosis as well as treatment of CRC.

## Introduction and Background

Although the incidence of colorectal cancer (CRC) is attributed to both hereditary and sporadic factors; the World Health Organization estimated approximately 30% of cancer mortality is attributed to adjustable risk factors such as life factors which includes consumption of alcohol, tobacco smoking, physical inactivity, and dietary habits (1). The world-wide mortality rate attributed to CRC exceeds 50%, from that of incidence (2); with over 1.8 million cases reported in 2018 (3). There is a well-established consensus on the link between the incidence of CRC (4) with high consumption of red and processed meat (5), and low consumption of dietary fiber based-foods (4) as well as the quality of dietary fatty acids (6), refined sugars, and alcohol beverages (7). There is substantial focus on red and processed meat (pork, sausage, beef, lamb, hamburger etc.), associated with the culinary techniques (8). It is considered that the application of high temperature to meat leads to the formation of compounds such as polycyclic aromatic hydrocarbons and polycyclic amines (9), which are harmful and linked to colon carcinogenesis (10). Furthermore, other mechanisms related to the development of CRC involve “red meat in relation to the formation of N-nitroso compounds from nitrite, and reactive oxygen species (ROS) from heme iron in the stomach” (10).

Accumulating evidence shows that nutrients and bioactive components influence metabolic traits *via* epigenetics. The influence of diet on epigenetic modifications occurs over a lifetime, and these epigenetic mechanisms give insight to the crosstalk between phenotypic changes in metabolic syndrome (MetS) and dietary factors (11). Dietary components are considered to have the most impact on the sensitivity of humans to the transgenerational epigenetic influence of nutrition. Early years investigation showed the effect of food consumption in the health of offspring in a rat model (12). Thereby validating the transgenerational effect of diet in the demonstration of maternal dietary protein restriction that led to reduction in cerebral weight as well as total cerebral cell number in the subsequent (second) generation (11). The detrimental effect of nutritional restriction on traits *via* epigenetic alteration, can be overcome by full postnatal nutrition (11). Rats fed with 50% of standard chow (SC) showed epigenetic alteration for 50 generations due to maternal under nutrition. Thus, establishing the possibility of rectification of the adverse effect of maternal undernutrition. The feeding with *ad libitum* and SC was accompanied with restoration in higher fat mass and lower bone mineral density (BMD) in adulthood (13). Varying diets and dietary interventions, including high-fat diets (HFD) and caloric restriction (CR), plant derivatives, and bioactive nutrients have been linked to epigenetic changes which alter cellular signaling (14), and this may impact CRC development (15, 16). HFD have been demonstrated to impact on the expression of miR and methylation changes in F0 spermatozoa, with same occurrence in F1. Increase in methylation of Slc3a2 (gene responsible for encoding 4F2- a cell-surface antigen heavy chain) and the reduction in methylation of Mfsd7 (major facilitator superfamily domain containing 7) and Tbrg4 (transforming growth factor beta 4) were also attributed to HFD effect in F0 and F1 spermatozoa (17). Additionally, glucose tolerance was observed in female offspring but not in males.

Healthy dietary habits with attributes of natural components from food with active lifestyle can promote prevention, and even treat some disease processes. Dietary components such as quercetin, omega-3 polyunsaturated fatty acids (PUFA), curcumin, folate, vitamin D, have been reported to act as chemo-preventive agents in colorectal cancer (18). “Although the mechanisms underlying the role of food in preventing or favoring CRC are not fully elucidated, growing evidence indicates that at least some of them involve miRNA” (19). However, understanding the precise molecular mechanisms underlying the attributes of these natural substances is required, this area represents an effervescent research area. However, a view into the world of the colon without consideration of the resident microbiota will be an incomplete adventure that could result in myopic understanding of the dynamics of the colon in a pathophysiologic state. Normally, the colon harbors micro-organisms such as viruses, bacteria, and fungi. Microbiota, particularly bacteria such as *Bifidobacteria* have been reported and is believed to have a positive impact on the host digestion and the immune system, while pathogenic bacteria have been attributed to tumor initiation and promotion by driver-passenger pathogen model (20).

### Non-Coding RNAs

The eukaryotic genome has been revealed by high-throughput approaches to be transcribed with about 20,000–25,000 gene encoding proteins (21), leaving approximately 95% of the genome as un-translated ncRNAs (22). ncRNAs have recently been attributed to the regulation of biological processes, including cell proliferation, differentiation, migration, and apoptosis, as well as epigenetic processes and gene expression (23).

**Figure 1:**
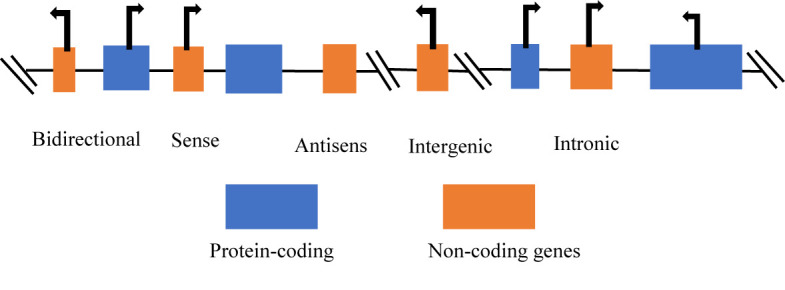
Categories of long non-coding RNAs (26). Long non-coding RNAs (lncRNAs) can be classified according to the position in the genome: bidirectional, sense, antisense, intergenic, and intronic lncRNAs.

The classification of ncRNA is categorically into two groups, with respect to size, such as small non-coding RNAs (sncRNAs), these are less than 200 nucleotides (nt) in length and the long non-coding RNAs (lncRNAs), which are more than 200 nt in length. The sncRNAs include microRNAs (miRs), small interfering RNAs (siRNAs), small nucleolar RNAs (snoRNAs), small nuclear RNAs (snRNAs), PIWI-interacting RNAs (piRNAs), and other sncRNAs. The lncRNAs are classified based on four major characteristics such as the genomic context and location, exerted effect on DNA sequences, targeting mechanism, and the mechanism of action (24). Furthermore, lncRNAs can be classified as bidirectional, intronic, antisense, sense, and intergenic (25) (**Figure 1**). The classification of lncRNAs depends on the relationship with protein-coding genes (27).

LncRNA are distinctive in their functional roles and gene expression with diversified functionality such as enhancers, decoys, guides, scaffold, and acting *via* genomic targeting, regulation in *cis* or *trans (* 28).

Non-coding RNAs can act as tumor suppressors or oncogenes in colorectal cancer and are considered potential diagnostic or prognostic biomarkers with potential clinical application. The advances in roles of ncRNAs in gastric cancer has been reported (26). Direct and/or indirect exertion of bioactive food ingredients affect carcinogenesis by impacting on cancer development *via* effect on gut microbiota composition/metabolism, and by regulating gene expression epigenetically. There is “complex interaction among food components and histone modifications, chromatin remodeling, DNA methylation, and ncRNA expression which leads to the regulation of gene expression and controlling of phenotype (29). High fat diet has been shown to impact on transgenerational metabolic phenotype through changes in miRNA expression, DNA methylation, and histone modification (30). The changes in DNA methylation in the intestine can be facilitated by microbiota-dependent regulation of intestinal cells. Microbiota can be affected by change in diet in terms of species relative abundance, this subsequently influences conversion of food components into metabolites which act as epigenetic regulators in cancer, as well as nutrient uptake and epithelial resilience (31). “Additionally, the complexity of inter-kingdom communication and its possible role in homeostasis and disease has been highlighted in the presence of cancer associated circulating miRNA” (32) and the attention to xeno-miRNA absorbed with food ingestion (33). Intestinal microbes such as probiotics (e.g., *Bifidobacteria*) consist of multiple layers of polysaccharide (PS), peptidoglycan, teichoic acid, and lipoprotein and these communicate with host immune system (34, 35). Majority of these molecules contain certain microbe-associated molecular patterns (MAMPs), which are specifically recognized by host intestinal mucosa *via* pattern–recognition receptors (PRRs) (36). The consumption of food such as dietary fiber, influences gut bacteria. This serves as energy source to both the colonocytes and favorable gut bacteria such as *Bifidobacterium* spp (37). Various daily dietary pattern exposes humans to xenobiotic electrophiles, such as cadmium (CD) from rice (38), and this can alter the redox and/or microenvironment (39).

### Dysfunctional Expression of MicroRNAs

MicroRNAs belong to the class of small ncRNAs. miRNAs are single stranded and ~22 long and are implicated in regulation of gene expression at post-transcriptional level (40). The resultant degradation or inhibition of translation by miRNAs occurs *via* the binding of miRNAs with a perfect or imperfect complementarity to the 3′-untranslated regions (3′-UTR) of a target messenger RNA (mRNA) (40). MiRNA can target numerous mRNA and each mRNA can be targeted by more miRNAs (41). Recent understandings of carcinogenesis have implicated miRNAs in all stages of carcinogenesis from initiation to the promotion and progression, *via* influence on differentiation, proliferation, apoptosis, angiogenesis, and metastasis (42). Furthermore, miRNA can be referred to as onco-miRNA (oncomirs), and act to reduce the expression of tumor suppressor genes, while tumor suppressing miRNAs can also increase the expression of oncogenes *via* down-regulation of the tumor suppressing miRNAs. There are inconsistencies to this account due to the limited understanding of the mechanisms underlying the specificity of oncomiRs (43). Reports have shown certain miRNAs to be oncogenic in a scenario and appears as tumor suppressive in another scenario. Therefore, cautious therapeutic approach is required. One of such miRNAs which act as oncomiR or tumor suppressor based on scenario is miR-125b. it acts as tumor suppressor in solid tumor but acts as oncomiR in most hematological cancers (43).

An Increasing body of evidence has shown the necessity of diet and biological dietary components for chemoprevention and treatment, based on miRNA modulation. miRNA expression in many diseases including cancer, is affected by bioactive food components (44). This review displays how dietary components can affect expression of miRNA and ncRNAs in colorectal cancer and thus influence carcinogenesis, tumor progression, and treatment.

### Oncogenic MicroRNAs

Aberrant expression of miRNAs in CRC can serve as tumor suppressors or oncogenes depending on the downstream target or connected signaling pathways (45). miRNAs such as miR-18a, -21. -31 and 92a, which are oncogenic are implicated in CRC development and progression.

The altered expression of miR-18a, which belongs to the miR-17-92 cluster, is associated with numerous cancers, including pancreatic cancer (46) and bladder cancer (47). The up-regulation of miR-18a was identified in 45 primary rectal tumor tissues compared to its adjacent normal tissues. MiR-18a was reported to play an oncogenic role in CRC *via* the downregulation of ataxia telangiectasia mutated (ATM); ATM was identified as a miR-18a target gene and a key player in repair of DNA double-stranded breaks. ATM is down-regulated in CRC tissues and inversely associated with the expression of miR-18a. The overexpression of miR-18a showed significant inhibition of repair of damaged DNA and enhanced etoposide-induced cell apoptosis, thus, it may be a potential biomarker for CRC (48).

### Colorectal Cancer and Small Interfering RNAs

Small interfering RNAs (SiRNAs) are in the class of sncRNAs, consisting of 21–25 nt which are formed by Dicer of the RNAse III family. The SiRNAs function as important members of sncRNAs, although not endogenously produced. Recently, there are siRNAs that have been identified as “star ncRNAs” in CRC. Cancer progression has been reported to have up-regulation of stromal interaction molecule 1 (STIM1) in highly invasive CRC tissues and cell lines, as well as the promotion of metastasis *in-vitro*, and *in-vivo*. Reversed effect was demonstrated *via* the silencing of STIM1 which is mediated by SiRNA. Additionally, epithelial-mesenchymal transition (EMT) inhibited *via* the silencing of STIM1 (stromal interaction molecule 1) was found to be a direct target of miR-185 (49). STIM1 is an endoplasmic reticulum Ca^2+^ sensor which triggers store operated Ca^2+^ entry activation. STIM1 is often synonymous with poor prognosis in CRC. The increased expression of STIM1 is indicative of metastasis and the progression of CRC. Human CRC cells show the expression of leukemia inhibitory factor (LIF), in hypoxic condition, that is mediated by HIF2α. The utilization of siRNA to knockdown endogenous HIF2α, inhibited by LIF induction by hypoxia (50). Colorectal cancer cell lines and tissues have also been reported to have up-regulated peroxiredoxins (Prdxs). Prdxs are vital scavengers of reactive oxygen species (ROS). The inhibition of CRC cell growth has been demonstrated *via* siRNA-initiated silencing of the Prdx2 gene, thereby leading to an increased apoptosis induction and a decrease in cell proliferation, as well as endogenous induction of ROS. Also, Prdx2 is implicated in the regulation of Wnt/β-signaling pathway (51).

### Colorectal Cancer and Piwi-Interacting RNAs

The Piwi-interacting RNAs (PiRNAs) are 26–31 nt in length and are novel sncRNAs that interact specifically with P-element-induced wimpy testis (Piwi) protein. PiRNAs can silence and control the transposable elements (TEs) and protect the genome (52); the loss of genome integrity can result from an uncontrolled expression of TEs.

PiRNAs play pivotal roles in human carcinogenesis (53). Significant up-regulation of PiR-651 in colon cancer and gastric cancer tissues have been identified (54). The binding of piRNAs to Piwi have been identified to determine CRC risk, and genetic variants in piRNAs modulate the susceptibility to CRC. The risk of CRC in association with the identified seven piRNA SNPs have been reported (55). The decreased risk of CRC showed significant association with SNP rs11776042 in piR-015551.

### Colorectal Cancer and Long Non-Coding RNAs

The advent of high-throughput microarray assays and bioinformatic approaches have revealed the expression of lncRNAs in CRC. lncRNAs are vital potential biomarkers or therapeutic targets, due to the close association between de-regulated lncRNAs and diagnosis and prognosis of CRC patients (56). There has been identification of 1,133 lncRNAs in metastasis lymph nodes (MLNs) compared with normal lymph nodes (NLNs) (57).

### Noncoding RNAs and Extracellular Vesicles

The recent advances in research on tumorigenesis has shed more light on cell to cell communication. Extracellular vesicles (EV) are involved in transferring non-coding RNAs in cell to cell communication; EVs are membranous small lipid bilayer vesicles secreted from various cell types (normal/diseased) and accumulating evidence has shown the importance of EV-associated non-coding RNAs in diseases such as cancer. EV-associated ncRNAs regulate the protein expressions of pivotal genes (58). Extracellular vesicles are differentiated into three main types based on their biogenesis and size; namely: exosomes which are released by multivesicular bodies are 40–100 nm in diameter, micro-vesicles which are 50–1,000 nm, are formed *via* direct budding from plasma membrane, while apoptotic bodies that are formed by apoptotic cells, are 800–5,000 nm in diameter (59).

Recent years have shown the loading of ncRNAs on exosomes. Depending on the target mRNA, EV-associated ncRNA function to both suppress and promote tumorigenesis and development (58). The existence of heterogeneity in tumor cells confers varying potential for proliferation, invasion, and metastasis, thus tumor cells cooperate to drive cancer *via* intra-tumor transfer of ex-miRNA (exosomal/extracellular miRNA). Stromal cells receive ex-miRNA in the local microenvironment or at distant sites to promote cell survival and the generation of pro-metastasis niche for colonization (60). Exosomes are not only endogenously originated but can be obtained from dietary sources, such as the transfer of bovine milk exosomes across species. Dietary miRNA encapsulated in milk exosomes are protected from low pH degradation, RNase, and treatment which mimics gastrointestinal (60).

### Diet and Microbiome

The upper and lower parts (oral cavity and large intestine) of the human digestive tracts are inhabited by a complex ecosystem of bacteria, viruses, fungi, and protozoa that are categorized as microbiome (61). This ecosystem begins to form before birth (in the Uterus) and develops after birth for 2–3 years to become stable and full functioning microbiome. Significant shifts in composition occurs due to physiological changes associated with senescence (61, 62). The occurrence of fungi and bacteria in both the oral cavity and the large intestine differs owing to the distinct epithelial layer and different oxygen levels. Approximately 10 (11) bacteria from the upper part get injected daily, into the lower part, and bacteria from the oral cavity overlaps with intestinal microbiota in ~45% of tested individuals (63). Anaerobic strains that inhabit the oral cavity have been implicated in oral diseases and are present in gut inflammatory and tumor tissues (61). The mouth maintains a constant humid and stable temperature between 34 and 36°, and due to varying pH level and the different types of diet; constitutes “the most diverse microbial communities due to the heterogeneity and the interrelationships between anatomic structures’’ (61, 64). The differences in composition and function of the microbiota of the digestive tract has been reviewed (65). Advance molecular methods have shown the involvement of Fusobacteria and other oral bacteria in pathological conditions including IBD and CRC (61). Accumulating evidence has established that the occurrence of colorectal cancer is associated with dysbiosis of the human gut microbiota as well as the formation of polymicrobial biofilms (66). Numerous studies have also recently demonstrated the association of oral bacteria with CRC mucosa. A substantial part of the gut microbiome that is implicated in CRC, is particularly composed of oral bacteria and Fusobacterium (61). Bacteria species, such as *Fusobacterium, Bacteroides fragilis* are implicated in primary and metastatic human CRC (67). The microbiota genome encodes a greater number of genes (~100-fold) than the human genome, thereby enabling microbiota to use indigestible substrates from humans (68). Microbial metabolic activity results in products such as short-chain fatty acids (SFAs), vitamins and other compounds which are important for host metabolism and survival. The host-microbiome interaction and/or sensing is important for appropriate mucosal function and immune response development and homeostasis. The release of small ncRNAs in the gut contributes to shaping the gut microbiota (69). The cross-talks that exist between gut microbiome and host are mediated by metabolites, proteins, and small RNAs (sRNAs), and this has been extensively reviewed and demonstrated by Tarallo & colleagues (69). The alteration of intestinal *Mus* musculus miRNA (mmu-miRNA) facilitates dysbiosis in the gut. The uptake of specific human sncRNA (hsa-sncRNAs) by *Fusobacterium nucleatum* and *Escherichia coli* have been implicated in regulating the expression of microbial genes to affect their growth. Also, perturbation of the gut microbiome and specific bacteria species have been reported to alter the expression of homo sapiens miRNAs (hsa-miRNAs) (70, 71). CRC-associated gut microbiome signatures among varying populations can serve as a new and accurate oncobiome-based diagnostic tool. Human and microbial sRNAs in fecal samples of patients with colorectal carcinoma or adenoma were compared with healthy subjects (69).

The exhibition of extracellular vesicles is a universal feature of metazoan cells, as well as prokaryotes (72). Bacterial microvesicles (bMVs) are underestimated in tumorigenesis. EVs from host, microbiota, and edible plants in the intestinal ecosystem are considered to act as key facilitators of intracellular and interspecies communication, and possibly promote tumor survival and multi-drug resistance (73). The increased levels of EVs in bile, plasma, urine, liquor, semen, saliva, synovial fluids, pleural, vitreous, intestinal fluids, and mucus, are often associated with the activation of cells and pathogenesis. Recent studies have focused on the communication between commensal, pathogenic microbial, immune system, and eukaryotic cells (74). Gut microbes communicate with their host *via* the epithelium both beneficially and/or pathologically in disease onset and progression. The differential signature expression of hsa-miR-21 as gut microbiome composition between adjacent colonic mucosa and CRC tissue showed the activation of toll-like receptor 4 (TLR4) signaling pathway in the CRC cell lines incubated with *F. nucleatum (* 73). The upregulation of *F. nucleatum* sRNAs and miRNAs of Epstein-Barr virus support the idea of host-gut microbiome sRNAs reciprocal interaction (74).

### Diet and Gut Virome

The viral component of the gut microbiome is referred to as the gut virome. There are eukaryotic viruses that can replicate in human cells, as well as bacteriophages that replicate in gut bacteria (75). Furthermore, the intake of food such as plants (76) and animals (77) are sources of eukaryotic viruses. Diet is a vital and constant environmental and lifestyle factor that impacts on the gut microbiome, including viral components (78). The complexity of Microbial-host homeostasis has been established with less consideration to the interplay between bacteria and virome components (78). The intestinal virome which is made of the DNA and RNA viruses exist in equivalent number to bacterial cells but may outnumber bacterial cells on gut mucosal surface within the mucus layers, to as great as 20:1. The human gut content per gram contains ~10^8^–10^9^ virus-like particles (VLPs) (78). The occurrence of eukaryotic viruses correlates with age of individual, such that eukaryotic viral population is low in infants but expands thereafter (79). Also, studies have proposed that the genetic constituent of an individual’s virome is a function of the genome, lifestyle, and behavior of that individual, with pivotal role played by age and medication (80). There are few studies on the impact of diet on gut virome. Although it is established that the density of gut bacterial changes over time because of environmental and inherited factors. Controversially, the gut virome is moderately stable within an individual. There is a discrepancy in findings on gut virome and diet. Reyes and colleagues reported the existence of a large variance between individuals on similar diet (81), Minot and colleague reported contrarily that diet has effect on the gut virome (82), while Kim and colleagues reported the significant occurrence of Caudovirales order in the mucosal and luminal of obese mice fed with high fat, and high sucrose “Western” diet (83).

The human gut virome is composed of animal viruses *via* oral route transmission by consumption of contaminated food and can be a source of infection such as the hepatitis E virus (HEV) that is responsible for acute hepatitis in humans (75). Although type 1 and 2 are specific to humans, pigs serve as reservoirs for type 3 and 4, with long-term infection described in immunocompromised patients. The first gyrovirus in human, belonging to the *Circoviridae* family, was initially seen on the skin of healthy people; and similar viruses are prevalent in chickens and human stools; this is seen as passive transmission of viruses and the replication of these viruses in humans *via* food intake (75). Gyro viruses express apoptin gene which encodes a protein that is specifically cytotoxic for cancer cells, this natural infection is specifically beneficial in controlling the development of tumor cells specifically in colon cancer (84). Numerous circoviruses and related single stranded DNA (ssDNA) that encode rolling circle replication initiator protein (Rep) have been characterized from tissues of mammals, plants (geminivirus and nanovirus), insects animal and human feces, algae cells as well as varying environmental samples (85). The genus *Cyclovirus*, is new in the *Circoviridae* family, with small circular ambisense genomes of 1.7–1.9 kb, and two major ORFs (inversely arranged), that encode putative Rep and Cap proteins. The pathogenicity of many Rep remains undetermined. Li and colleagues demonstrated the presence of *Cycloviruses* in 40 of 395 (10%) of Nigerian, Pakistani, and Tunisian human fecal samples (86, 87), and in 6 of 44 (13%) wild African chimpanzee fecal samples. Furthermore, the *Cycloviruses* were detected in 22 of 40 (55%) Nigerian chicken samples, 7 of 51 (14%) Pakistani and Nigerian beef samples, 8 of 73 (11%) of Nigerian sheep and goat samples, and 3 of 27 (11%) of Nigerian camel samples (87, 88). The interplay between virus and bacteria impact on host health and disease (78). The antiviral immunity in human rely on Gram-negative commensal-dependent NF-κB signaling, while enteric viral infection shields against intestinal damage and pathogenic bacteria. This adds to the complexity of the host-microbe homeostasis (89).

### Human Colonic Mycobiota

The complexity of the intestinal ecosystem is not limited to the presence of bacteria and viruses. Fungus are majorly less researched in relation to colorectal cancer with extremely limited literature to show the implication of fungus in colorectal cancer. The ubiquitous fungus in nature are the *Pneumocystis* spp. and *Aspergillus* spp. which are the cause for fungal infection worldwide. Also, of worldwide distribution and endemic are the *Scedosporium* spp. and *Cryptococcus* spp.; *Scedosporium* spp. have been identified in soil, ponds, tidal flats, manure, and swamps, while *Cryptococcus* spp. was identified in pigeon droppings. *Candida* spp. are commensals of the gastrointestinal tract (90). *Cryptococcus* spp. and *Aspergillus* spp. are recognized as integrant of the human colonic mycobiota (91).

Entomophthorales and Mucorales, which are two orders of the fungal subclass Zygomycetes, are endemic fungi. Entomophthorales and other spps such as *Penicillium* spp., *Histoplasma* spp., and *Paracoccidioides* spp. have been identified in soil of some regions of the world (90). Foods like bread, fruits, and other organic substrates including vegetable matter, animal excreta, soil, and compost have been reported to constitute Mucorales, worldwide (92).

There exist extremely limited studies on the role of ncRNAs in fungal infection. Little is known on the regulatory impact of ncRNAs in fungal infection. The infection with *Arabidopsis thaliana* has been reported to implicate lncRNAs and long intergenic non-coding RNAs (lincRNAs) in antifungal immune response (93). There are implications of several miRNAs in respiratory epithelium during infection with *Candida (* 94). Recently, using RNA-Seq, Riege & colleague demonstrated the involvement of lncRNAs in humans during fungal infection and the effect of vit A and D during fungal infection (95). Also, the implications of ncRNAs in several functions in yeast have been identified; four essential ncRNAs were identified, namely, SUT527, SUT075, SUT367, and SUT259/691 (96). The effects of each new ncRNA on adjacent gene expression in heterozygote background identified both induction and repression of nearby genes. The use of plasmid complementation showed rescue of SUT075 lethal phenotype, to reveal the involvement of the ncRNA in *trans (* 96). The role of ncRNAs in fungus during colorectal cancer development and progression and its interaction with components of the intestinal ecosystem has not been described.

Fungal EVs carry known virulence factors such as polysaccharides, lipids, RNA, proteins, and pigments and this supports the hypothesis that the fungal EVs concentrate pathogenic determinants (97). Fungal EVs stimulate host immune system.

### Protozoa and Human Gut

The saying goes that “you are what you eat,” this saying can be rephrased as you are what you eat and where you live (resident). Industrialized countries are considered to have less diverse population of gut microbiome, compared to underdeveloped/non-industrialized countries, This diversity results from the variation in antibiotic and dietary fiber intake, as well as food sterilization. It has been proposed that decreased prevalence of helminths and gut protozoa could partly account for the diversity loss (98). There has been limited attention given to the occurrence of protozoa as part of the gut microbiota. This is partly due to the limitation on the approach in amplifying the 16S rRNA gene and based on the research outcomes in industrialized countries with low infectious burden (98). Industrialized countries have decreased exposure to environmental microorganisms and, mainly due to sanitation improvement, there by reducing horizonal transmission of microbes (99). Protozoa have previously been recognized as parasites with detrimental effect to host. The known protozoa pathogens in humans are *Cryptosporidium* spp., *Entamoeba histolytica, Giardia intestinalis*; however, important protozoa like *Blastocystis* spp. are prevalent in healthy populations and are categorized as members of healthy microbes (100). Protozoa were recognized as part of human ancestral gut. Evidence provided by lateral gene transfer (LGT) confirms the coevolutionary history of these organisms, with the acquisition of approximately 2.5% of genes in *Blastocystis* spp. by LGT from their donors which are mostly bacteria (101). Certain health conditions have implicated *Blastocystis* spp. as causative agent, where it is considered as the infectious agent, presenting symptoms such as diarrhea, abdominal pain, anorexia, nausea, bloating, and vomiting (102). Contrarily, healthy subjects have shown higher occurrence of *Blastocystis* spp. compare to ulcerative colitis and irritable bowel patients (103). The interactions which indicate the clinical relevance of *Blastocystis* remains unclear. There are approximately 17 *Blastocystis* genetic subtypes, and the correlation between pathogenicity and subtypes has been hypothesized for long, with inconclusive outcomes (98).

### Dynamics of Relationship in the Intestinal Ecosystem

There are several hypothetical models that have been hypothesized to be responsible for phage driven-dysbiosis in the intestine: the “kill the winner” model involves the phage targeting and killing dominant commensal bacteria which are usually growing fastest (104). Another mechanism involves the “biological weapon” model where commensal bacteria utilize the phage they possess as weapons to kill competing bacteria, thus leading to dysbiosis; this model may favor a protective role against pathogens, however, there are limited experimental evidence (105). Additionally, “community shuffling” model states that environmental stressors such as antibiotic therapy, inflammation, or oxidative stress results in the introduction of prophage into bacteria, which leads to lytic infection of symbiotic bacteria and alter the relationship between symbionts and pathobionts (82). Furthermore, the “emerging new bacterial strain” model involves the ability of phage to transfer genes to bacteria to modify their phenotype, thereby establishing lysogeny rather than lysing (82). The driver of this differential behavior is oblivious.

### Colon Defense and Immunity

The human tissue is equipped with a defense and immune system that protects the cells and tissues from damage. Cells and Tissues have various types of defense mechanisms against various attacks poised at it. Defense and immunity are responses to foreign and/or unusual activity within the cell/tissue environment. The presence of foreign substances, toxins, or metabolites results in imbalance activity within the cell and/or tissue environment, thereby eliciting defense mechanisms such as the release of stress proteins in response to stress. The intestinal environment is characterized with reciprocal and dynamic interactions between the epithelium, microbiota, and mucosal immune system. This interaction involves complex elaborated regulatory mechanisms to maintain symbiosis and prevent aberrant responses that lead to pathological states (106). All cancers develop through the accumulation of epigenetic and genetic mutations (15). Defense and immunity are a paramount occurrence in all cells and tissues, especially in the colon, particularly, due to the involvement of the colon in food digestion and reabsorption. Defense and immunity are affected by the diet of an individual (107). Bioactive food produces oxidative stress that results in DNA damage. There is a growing body of evidence that supports the involvement of nitroso compounds (NOC) and heme iron as being fundamental in colorectal carcinogenesis (108). NOC are formed *via* the action of nitrogen oxides or nitrite with secondary amines and N-alkylamides, to yield N-alkyl nitrosamides and N-alkyl nitrosamines. The formation of NOC in the large intestine is majorly dependent on dietary type and bacterial colonization, which are main factors of colon carcinogenesis. The formation and occurrence of NOC has been extensively reviewed by Fahrer & colleague (109). The defense and detoxification of NOC is carried out by the suicide enzyme; O^6^-methylguanine-DNA methyltransferase (109). The O^6^-methylguanine DNA methyltransferase (MGMT) is responsible for the resistance to DNA-alkylating agents in normal cells, and its mostly at increased levels in tumors, relative to normal cells (110). MGMT repairs genomic DNA. O^6^-Methylguanine pairs with thymine, leading to a G- to-A transition during DNA replication if left unrepaired. Epigenetically silencing of MGMT occurs in various tumors, including colon cancer (111, 112). Colorectal cancer case shows association of MGMT promoter methylation with G-to-A mutations in p53(26) and in KRAS (113). The protection of the genome from reactive oxygen species is mainly carried out by base excision repair pathway (110).

Prior to genomic damage, the defense against oxidative stress which is a consequence of production of free radical is primarily combated by glutathione detoxification system. Studies have demonstrated the involvement of the gut microbiota in the digestive and immune system as a source of regulatory signals which influence development and maturation. Outer membrane vesicles (OMV) have been shown to be how host-microbiota communicate in the defense and immune system of the colon. The OMV enable microbiota-interaction by mediating signals and events to the immune system *via* intestinal epithelial barrier. Exosomes contribute to innate immunity in both plants and animals (114).

### Immunity Against Infection

The type of diet of an individual determines the species of occurring microbiota in the colon of the individual (as shown earlier in this review). The host beneficial microbes thrive on undigested fiber residue in the lumen and yield metabolites that function in a complex metabolic network and are inhibitory to pathogenic organisms. The presence of non-beneficial microbiota results in induction of the immune system. The Complement cascade which effectively links the innate and adaptive immune response, is an important component of innate immune response that is responsible for the neutralization of microbes and the clearance of cancerous cells; and directly lyses infected and/or abnormal cells as well as pathogens (115, 116). The complement cascade consist of three pathways: classical, lectin, and alternative pathways (AP).

The immune response to fungi may play an integral role in pathophysiology and pathogenesis of colonic infection; this immune response is from the human and accompanying microbiota component of the intestinal ecosystem (117). The elimination of fungus by the immune cells is usually *via* soluble and membrane-bound receptors on innate immune cells. Lectin, a membrane bound receptor, as well as complement receptors and toll-like scavenger, can activate phagocytosis and respiratory burst *via* detection of fungal products and fungus. The membrane-bound receptors also induce proinflammatory cytokines and chemokines *via* the activation of transcription factors. Additionally, the triggering of T helper (Th) 1 and 17, as well as the production of other cytokines such as interleukin (IL)-17A, IL-17F, and IL-22 for adaptive immunity are host defense against fungi (117). The immune status of an individual is considered a predisposing factor to colonic fungi infection. The increase in colonic infection by fungi can result from impaired production of IL-22. The increase in fungal burden by defective IL-22 and IL-23 pathway have been reported. Innate cells produce IL-22, which is regulated by IL-23 and activate inflammatory cells to control fungal growth (118).

Glutathione is the major determinant of cell redox status, playing a key role in stress response in living organisms (119). Glutathione influences defense, secondary metabolites, protein activity regulation *via* conjugation reactions to metabolites or to protein cysteine residues (120). The protection of immune cells and the formation and proliferation of new immune cells is by glutathione. The production of white blood cells, particularly T cells (lymphocytes) to fight infection is boosted by glutathione. Healthy immune system anchors on well-functioning and healthy lymph cells infused with balanced glutathione level. The changes in the level of glutathione affects functions of the lymphatic system. Studies have demonstrated glutathione to be the food that boosts the strength of the lymphocytes. Abundance of glutathione supply is required for the protection against microorganisms and viruses (121). Researchers have reported the dependence of all cellular functions including DNA and RNA synthesis on sensitivity to reactive oxygen and peroxides, which both rely on healthy levels of glutathione. Cysteine and methionine are amino acids that aid in RNA and DNA synthesis as well as in glutathione. The immune system must be exhausted in-order for cancer to initiate and progress. The exhaustion of the immune system relies on the availability of glutathione because the lymphoid cells function best on a balanced level of glutathione (122).

Glutathione can be found in foods such as grains, fresh fruits, vegetables, carbohydrates, and fish (123). Polycyclic aromatic hydrocarbons and heterocyclic aromatic amines are known risk factors of colorectal cancer development; and glutathione-S transferase is involved in the detoxification process of both types of aromatics. Polymorphism in GST is often associated with alteration or absolute lack of enzyme activity (124). Kim & colleagues reported that the levels of GSH and GSH synthetic enzymes in human colorectal cancer have shown that mRNA and protein expression of GSH, the catalytic subunit of GCL (GCLC), and GSS were significantly upregulated in five colon cancer cell lines: SNU-407, Caco-2, SNU-1033, HCT-116, and HT-29, when compared to the normal colon cell line, FHC (125). Patients with colon cancer have shown higher expression of GSH levels in tumor tissues of 9 out of 15 cases, compared to adjacent normal tissue. Additionally, GCLC and GSS protein expression levels have shown to be higher in tumor tissue of 8 out of 15 and 10 out of 15 patients with colon cancer, respectively. Furthermore, a report on polish population demonstrated the association between GST genotype polymorphism and CRC development in carriers of GSTT1-null and GSTP1-variant genotypes (124). Was & colleagues demonstrated the higher but non-significant (45.9% *vs*. 42>9&; p>0.05) prevalence of the GSTM1*0 variant in CRC patients than in control subjects. The frequency of GSTT1*0 variant was also reported to be higher in CRC patients compared to the control population (21.1% *vs*. 18.9%: p>0.05). Associating the incidence of CRC for GSTM1*0 and GSTT1*0 variants showed a not statistically significant difference for increased risk of CRC. However, examining the GST genotype in relation to tumor-node-metastasis (TNM) classification and primary tumor location demonstrated a significant difference of the GSTT1*0 and GSTT1*1 in both location and stage of the primary tumor (126). However, the mechanisms of regulation of GSH metabolizing enzymes by diet in human colorectal cancer cell lines and tissues remains unelucidated. The influence on GST polymorphism and the intake of red meat on CRC occurrence have been reported in Polish population. Klusek and colleagues reported that in the absence of polymorphism, the analyzed genes showed no association between a high frequency meat consumption and the occurrence of CRC. However, in GSTM1 gene polymorphism, high frequency of meat consumption increased risk of CRC by about four times (OR adjusted for sex and age=3.8, 95% CI: 1.6–9.1), while GSTP1 gene polymorphism showed a three-fold increased risk of CRC with high frequency of meat consumption (OR adjusted for sex and age=3.4, 95% CI: 1.4–8.1). GSTT1 gene polymorphism showed non-significant increase in CRC risk (OR adjusted for sex and age=1.9, 95% CI: 0.4–8.5). Additionally, the risk of CRC increased in non-smokers with GST gene polymorphism that are on high frequency intake of red meat (124).

### The Protective Effect by Diets

Chemo preventive effect is exhibited by some food components such as short chain fatty acids (SCFA), polyphenols, and omega-3 PUFA (127). Polyphenols are ubiquitous secondary metabolites, occurring in vegetables and fruits, beverages (wines, tea, coffee), and whole grain cereals. Resveratrol (RES) and quercetin are polyphenol containing-food. The occurrence of resveratrol in peanuts, dried fruits, berries, and grapes influence the level of miR targeting both tumor suppressor and oncogenes. The increase in levels of tumor suppressor gene-miR663 by RES influence the target TGFβ1 transcript (128). Also, the anti-proliferative/proapoptotic influence which targets ERK/c-Myc *via* miR-143, is exhibited by α-mangostin (α-M), a xanthone from mangosteen pericarps (129). The combination of phytochemicals (quercetin, RES, epigallocatechin-3-gallate, and α-M) or phytochemicals with anti-cancer drugs such as 5-fluorouracil (5-FU) has been shown to have a pro-apoptotic effect on CRC cells (130). One of the possible mechanisms is the repressing of Sp-1 activity *via* the interplay of RES and quercetin with miR-27a-ZBTB10 axis. The up-regulation of miR-34a and the down-regulation of its target genes E2F3 and Sirt1, was identified as a chemo-sensitizing activity of the combination of EGCG and RES, which led to promoting apoptosis. The synergistic effect on growth inhibition by α-M and 5-FU has been reported (129). Furthermore, observation of colon cells showed the inhibition of reactive oxygen species (ROS) and NF-κB activation *via* the induction of miR-126 and miR-146a (131). *In-vitro* and *in-vivo* reports on pomegranate (PO), demonstrated significant changes in cancer markers and p21waf1/Cip1 (CDKN1A) induction as a mechanism underlying anticancer effect of urolithin-the gut microbiota derived metabolites of PO (132); the up-regulation of miR-215 or down-regulation of miR-224, was implicated in CDKN1A induction.

Additionally, the *in-vivo* report on RES and proanthocyanidin-rich extracts showed prevention of tumorigenesis *via* miRNA modulation in the suppression of inflammatory pathway and Kras activity (133). Also, experimentally induced colon carcinogenesis in a rat and CRC cells showed interplay of the PO with miR-126/VAM-1 and miR-12/PI3K/AKT/mTOR, this is identified as a mechanism which in part mediate anti-proliferative and anti-inflammatory activity of PO polyphenols (134). However, controlled human trials showed effect on specific miRNA other than miR-126 in the consumption of PO (135).

### Noncoding RNAs and Extracellular Vesicles of Plants

Extracellular miRNAs occur in dietary plants. It is suggested that dietary miRNAs transfer to the systemic circulation *via* the guts to impact biological effect on animal physiology. Currently, the release of miRbase 2018 version (v22) states that humans have 1917 precursor and 2654 mature miRNAs. “*Arabidopsis thaliana*, the model organism for plant biology” (136) has 326 precursor and 428 mature miRNAs; however, biogenesis of miRNAs in plant kingdom differs significantly from animals (136). Crops such as wheat, soybean, and rice possess 122, 684, and 604 pre-miRNAs and 125, 756, and 738 mature miRNAs, respectively. Accumulating literature have suggested pivotal role of plant miRNAs in stress response, development, and disease resistance in plant (137). The focus of this review is on the influence of dietary ncRNAs in human health, specifically in CRC. Although considered as fairy tale, recently, cross kingdom regulation of human health by dietary plant miRNAs is gaining attention. Although controversial, dietary sources of miRNA was reported by Zhang & colleagues (138), demonstrating the presence of hundreds of miRNAs in serum samples of healthy volunteers, thereby revealing the stable nature of miRNAs in extracellular environment as naked duplex proteins or in encapsulated vesicles such as exosomes. MiR-156a and miR-168a were identified in Chinese cohort and rice derived miRNA-168a was reported to target human mRNA low density lipoprotein receptor adaptor protein 1 (LDLRAP1) in liver of human/mice (137). The question of the limiting barrier of the intestinal tract being an obstacle for the transfer of miRNA from diet, was addressed in systemic delivery of siRNAs to *Caenorhabditis elegans.* Oral feeding with *E. coli* showed over expression of specific gene of siRNAs, thus indicating that orally delivered sRNA can pass through the GI barrier (137). Further studies reported identification of small RNA from next generation sequencing of human serum samples, the notable sRNA was from rice and corn. Hirschi & colleagues reported plant miRNAs in sera and plasma of mice fed with honeysuckle (HS) diet. High level of miR-2911, was observed within 3.5 days of post-feeding in mice serum and urine; miR-2911 is derived from 26S ribosomal RNA. Also noted was the influence of dietary miRNAs in absorption; combination of miR-168a with HS compared to feed of miR-168a alone resulted in increased plasma level of miR-168a (137).

There are very few studies on plant exosomes. These studies have shown the defense exhibited by multivesicular bodies in plant cells, during pathogen attack (139). The secretion of exosome in plants is implicated in early defense response to pathogens (140). Additionally, exosomes have been implicated in the transport of several compounds and proteins, and are known to mediate the transport of small interfering RNAs from plant cells into fungal cells, thereby inhibiting the expression of target transcripts in invading fungus (141); although the origination of the vesicle between the plant and the fungus is unclear (142). EVs are enriched in stress and defense related proteins including PEN1. The infection with virulent strain of pathogens such as *Pseudomonas syringae* enhances the secretion of EVs, and this secretion has also been shown to be enhanced in response to salicylic acid (SA) (114).

### Nutrient Signaling, Metabolism, and Colorectal Cancer

Unhealthy diets can alter gene expression. Abnormal miRNA profile that is synonymous with nutrient surplus is recognized with metabolic syndrome diseases such as cardiovascular diseases and type II diabetes (T2D). Recently, food-borne miRNAs are drawing the attention of scientists showing that exosomes function as transporters to transfer nutritionally derived miRNAs into human objects (64). mTORC1 is a nutrient sensor that influences cell growth and proliferation *in-vivo*. Nutrients, stress signals such as hypoxia and glucose deprivation, growth factors (GFs) as well as cellular energy status are upstream signals that impact on mTORC1 activity (143). The activity of mTOR1 is essentially dependent on the integration of GF and nutrient signals. The activation of mTORC1 impact on cellular processes, including nucleotide, protein, and lipid synthesis, cellular metabolism such as ATP production, and autophagy, thereby functioning to maintain balance between anabolic and catabolic processes (143). Activities such as translational machinery that promotes growth and proliferation through the phosphorylation of ribosomal protein S6 kinase (S6K) and the eukaryotic translation initiation factor 4E(elF4E)-binding protein (4EBP). mTORC1 plays important role in lipid biosynthesis through SREBP ½, thus, increasing the glycolytic flux by activation of transcription and translation of hypoxia inducible factor 1α (HIF1α), and regulating the expression of genes associated with oxidative metabolism, and promoting growth by inhibition of autophagy and lysosome biogenesis. Therefore, mTORC1 is recognized as a critical sensor of nutritional cues and an important player in maintenance of intestinal epithelial homeostasis. Intestinal stem cell (ISC) is regulated by mTORC1 in Paneth cells, in response to nutrient availability (143). The past decade has witnessed work on the involvement of ncRNAs, specifically long ncRNAs (lncRNAs) in lipid metabolism, acting mostly at transcriptional level. miRNAs such as miR-122, miR-33, and miR-143a have been reported to play critical roles in controlling cardiovascular disease *via* the regulation of cholesterol homeostasis and metabolism (144).

### Influence of Diet on Hereditary Colorectal Cancer

Hereditary CRC makes up approximately 2–5% of all CRC. The implication of glutathione level, function, and regulation on hereditary predisposition to CRC has not been an area of much investigation. Accumulating evidence that show the implication of glutathione level and GST polymorphism in the development of CRC, will warrant questions on the interplay between the varying hereditary disposition of CRC and glutathione components. The *in-vivo* mouse model study on the relationship between APC gene and glutathione conjugating enzyme variant such as GSTP1, showed a six-fold increase in incidence of colon adenoma in *Apc* ^Min/+^ *Gstp1/p2* ^−−/−−^ (Gstp-null *Apc^Min^*) than in *Apc* ^Min/+^ Gstp1/p2^+/+^ (*Gstp*-wt *Apc* ^Min^)-the wild counterpart that rarely develops colon cancer (145).

### Influence of Diet in Other Cancers

Accumulating evidence continue to enlighten on the pivotal role of diet in health and disease prevention through the mode of action of phytochemicals. The consumption of certain food such as curry spices, red grapes, soy, and blueberries have been shown by epigenetic diet research to have beneficial effect on prevention of cancer (146, 147). Furthermore, the dietary impact on epigenetics is considered to extend to the unborn child (*in utero*) of individual consuming epigenetic diet, thus resulting in epigenetic profile reprogramming, thereby altering the predisposition to diseases such as cancer (147). The influence of diet on epigenetic profiles is achieved *via* modulation of ncRNA, particularly miRNAs (148). Vesicles containing miRNAs have been detected to be released from breast cancer (BC) cells, and this along with the findings of miRNAs in milk, blood, and other body fluids, underlines the necessity for investigating miRNAs and lncRNA in the circulation (149, 150). Recent research advancement has also shown the indication of delivery of food derived exosomes/vesicles (FDE) to other organs through the blood stream and still able to function distantly in recipient cells (151). The mechanisms entailed in the FDEs packaging and uploading of cargo is unclear, but the physiological and pathological controlling process of FDE as a therapeutic vehicle continues to gain the attention in drug design and delivery. The suppression of prostate cancer by the impact of diet on lncRNA expression have been shown *via* the activities of sulforaphane (SFN) from vegetables such as broccoli. SFN has been demonstrated by Beaver & colleagues to attenuate the expression of lncRNAs associated with cancer formation. The over expression of LINC01116 in several cancers have been shown to be transcriptionally repressed after SFN treatment (152).

### Exogenous Exosomes

The knowledge of exosomes being a communicating tool between cells has given rise to the possible use of exosomes as therapeutic tools for drug delivery. Originally, exosomes were thought to be “garbage bags” for the disposal of irrelevant cellular components, growing evidence accept the usefulness of exosomes as vehicles for drug delivery molecules in similar structure to liposomes (153). Exosomes are now recognized as involving in specialized intercellular signaling delivering nucleic acids, proteins, lipids, and metabolic cargos from source cells to neighboring cells and distant organs (154). Exosomes are regulators of cellular functions including apoptosis, proliferation, and migration, they are reported to be key players in tumor growth; secreted more in cancer cells to facilitate metastasis (155). Viral and cellular originating non-coding regulatory miRNAs, control gene expression *via* repression and translation of mRNAs into protein. miRNAs are secreted through vesicles called exosomes and are protected from degradation by RNases, this suggests that miRNA functions outside the cells that they are produced (156). Exosomes in recipient cells deliver cargos that facilitate gene expression and metabolism, such as in the role of miRNAs in the communication of regulating over 60% of mRNAs in humans. The functional delivery of viral miRNA was demonstrated by Pegtel & colleagues (156); miRNA by “EBV-infected cells were transferred to and shown to act in uninfected recipient cells. EBV-Infected B cells secrete EBV-encoded miRNAs, the EBV-miRNAs function due to internalization of exosomes by MoDC, resulting from dose-dependent miRNA mediated repression of confirmed EBV target genes including *CXCL11/ITAC*, immunoregulatory gene down-regulated in primary EBV-associated lymphomas” (156). The accumulation of EBV-miRNAs in non-infected neighboring MoDC throughout the coculturing of EBV-infected B cells demonstrated that the accumulation is mediated *via* transfer of exosomes, Therefore indicating that exogenous EBV-miRNAs transferred *via* exosomes are delivered subcellularly to sites of gene repression in recipient cells. Also suggestive of miRNA transfer is the demonstration in peripheral blood mononuclear cells from patients with increased EBV load, presence of EBV BART miRNAs in both B-cell and non-B-cell fractions despite restriction of EBV DNA in circulating B-cell population (156).

The loss of miRNA maturation in *Drosha* knockout mice leads to premature lethality. Adipocyte-derived exosomes are involved in the transforming growth factor-β in hepatocytes and obesity related liver disease (157). Mounting evidence have shown that exosomes and their cargos are not only derived endogenously but may be obtained from dietary sources, such as the transfer of exosomes of bovine milk across boundaries of species. Research has shown the transfer of bovine milk exosomes from the human intestine, vascular endothelial cells, and intestinal rat cells into cytoplasm *via* endocytosis and releasing their miRNA cargos across basolateral membranes in cell cultures. Additionally, exosomes derived from pigs have been demonstrated to increase proliferation of intestinal cells, developing intestinal tract in mice (157). Exogenous exosomes have been demonstrated to be involved in promotion of intestinal cell viability, epithelial proliferation, and activity of stem cells. Li & colleagues reported the influence of rodent breast milk derived exosome, and further demonstrated the impact of bovine milk-derived exosomes on goblet cells. “The exposure of human colonic LS174T cells to exosomes in culture, promoted goblet cell expression *via* increased mucin production and relative expression levels of mucin 2 (MUC2) and trefoil factor 3(TFF3), goblet expression marker” (158). Additionally, treatment with exosome showed enhancement of expression of glucose-regulated protein 94(GRP94), which is the most abundant intraluminal endoplasmic reticulum (ER) chaperon protein involved in aiding protein synthesis (158). The study by Li & colleague also showed that exosomes administered to necrotizing enterocolitis (NEC)-an intestinal injury with impaired mucin synthesis, was able to prevent the morphological injury (158), thus showing the novel ability of exosomes in treatment of high-risk infants with NEC disease, whose very low birth weight and premature birth has mortality rate of approximately 50% due to extensive intestinal necrosis, and organ failure (158).

The insight on the responsibility of exosomes in cell-cell communication has shown that cancer cells secrete more exosomes than non-cancerous cells and that through the transfer of host cell’s invasive properties to non-metastatic cancer cells, cancer-derived exosomes promote metastasis *via* numerous ways including the alteration of the immune system, organotropism, promotion of EMT and angiogenesis, reviewed extensively by Zhang & colleagues (155).

Exosomes are advantageous compared to artificial nanoscale vehicles. The natural delivery made by exosomes to their membrane and cytoplasm components *via* fusing with target membrane, allows for exogenous therapeutics to be encapsulated in exosomes and deliver in a “hitchhiking manner” (155). Additionally, exosomes from patients’ blood or tissue, have low immunogenicity and have “long-term circulatory and excellent bioavailability” (155). Furthermore, exosome support targeted cargo delivery due to the specificity in cell types, exhibiting specific cell tropism (159).

## Discussion

This review presents the impact of diet on the critical role of occurring exosomes and ncRNAs in the intestinal ecosystem, and in colorectal carcinogenesis. The consumption of red meat causes inflammation of the diverticula of the colon and dietary fibers have been shown to reduce the risk of diverticulitis (160). Cholesterol stimulates inflammatory factors *via* toll-like receptors. Cholesterol is known to promote inflammatory response in the artery wall in genetically predisposed individuals due to increased level of low-density lipoprotein (LDL). The increased level of LDL in the blood results from the accumulation of cholesterol, due to high consumption of high fat or high fat cholesterol diet (161). The resultant metabolism of cholesterol is bile acid production (162). Dihydroxy bile acids cause mucosal permeability and result in uptake of bacteria in the human colon (163). EVs are important players in cell-cell communication, nutrition, and physio-pathogenesis, which are recently considered to be key mediators of immunopathogenesis in fungi, bacteria, and protozoa. Majority of research activity has focused on bacteria EVs, with little or no consideration to fungal and other intestinal microbes’ EVs. The sequence of events that results in cancer initiation and progression is pivotal and the understanding of these events will give insights into the prevention, diagnosis, and treatment/cure of the disease. The major negative influence of diet on the colorectal ecosystem is the production of free radicals and inflammation. These consequently impact on macromolecules that alter the microenvironment. Alteration in microenvironment leads to homeostasis disruption. Dietary components such as fat which results in increase in physiological level of bile acids and the action of bacteria to form secondary bile acids such as deoxycholic acid have been shown to alter the microenvironment through the production of free radicals, thereby causing hypoxic condition (our unpublished work). Hypoxic condition creates an environment that alters the normal physiological gene expression (164) and impacts on the inhabiting microorganism in the intestine. DNA damage to bacterial cells occurs (165), as well as impacting on the signaling activity involved in relaying messages to communicating cells. Although the mechanism of influence of hypoxic condition remains unelucidated in colorectal cancer; Chen & colleagues demonstrated the influence of hypoxia on hnRNPM. “Hypoxia increases cytosolic hnRNPM binding to its target mRNAs and promotes translation initiation” (164). The development of colorectal cancer is associated with elevation of hnRNPM and is associated with poor prognosis. The involvement of a unique set of hnRNPM-targeted genes as revealed by genomic-wide transcriptomic and translatomics analysis can be seen in metabolic processes and cancer neoplasia, translated selectively under hypoxia (163). Physiological conditions maintain tissue/organ homeostasis *via* the equilibrium between cell division and cell death. Mechanisms such as DNA repair and recombination, cell cycle checkpoints as well as cell death are related to this equilibrium. These mechanisms are influenced by the oxidation and reduction of proteins, alongside the nature and rate of free radicals’ generation. The state of cellular redox describes the balance of GSH/GSSG, NAD+/NADH, or NADP+/NADPH, as well as its relationship to the various sets of metabolites and the involvement in cell metabolism (166). Free radicals are highly reactive and cause damage to lipids, proteins, carbohydrates, nucleic acids, and result in loss of molecular functions (166). Reactive oxygen species (ROS) are the more abundant free radical in mammals and includes hydroxyl radicals, superoxide anions, and peroxide radicals. Other free radical molecules include the unpaired but harmful ROS-related hydrogen peroxide (H_2_O_2_), reactive nitrogen species (RNS) such as nitric oxide and peroxynitrite are essential regulators. Sources of ROS include the electron transport system, Krebs cycle, various oxidases (xanthine oxidase, NADH oxidase, arachidonic acid oxygenase activities), and the release of radicals from immune cells. The promotion of colon cancer by bile acid also involves the generation of reactive nitrogen that are cytotoxic and can result in DNA damage. The formation of nitrotyrosine residues in proteins was shown to be associated with plasma membrane of colonic epithelial cells, thus implicating bile acids as dietary risk factors for the direct generation of reactive nitrogen species in colonic epithelial cells, and it is independent of the RNS generated by immune cells (167). Washo-Stultz and colleague also indicated the induction of apoptosis in culture cells by bile acids and in normal colonic epithelial cells in human biopsy specimens. Also reported is the resistance to apoptosis induction by bile acids in normal-appearing flat mucosa of patients with colon cancer. The group hypothesized that resistance to apoptosis could arise from the selection of apoptosis-resistant cells. Apoptosis resistance is a survival phenotype that may contribute to genomic instability, enhancement of mutagenesis and tumorigenesis (167). The activation of poly (ADP-ribose) polymerase, a DNA repair enzyme by bile acids have been reported, that colon cells showed inhibition of reactive oxygen species (ROS) and NF-κB activation *via* the induction of miR-146a (131) and this enzyme has also been shown to serve as a protective role. Insufficient mounting of overall defense against stresses results in cells’ response to apoptosis. As reported, the possible action of bile acid induction of the target gene of NF-κB inducible NOS (167, 168) can lead to the inhibition of miR-126 and miR-146a. Therefore, the ability of some dietary plants/fruits to inhibit free radicals means the capability to influence ncRNAs in stress response and specifically in oxidative stress. Their mechanism could be the impact on the cis and trans pattern of the ncRNAs that are involved in oxidation/antioxidant system. Further investigation into the ncRNAs associated with GSH is required. Theoretically, oxidative stress is accompanied with the activation of antioxidant pathways. The nuclear factor erythroid 2-related factor 2/Kelch-like ECH-associated protein 1/antioxidant response element (Nrf2/Keap1/ARE) pathway is effectively activated as automatic response to oxidative stress for the maintenance of balance in oxidation/antioxidant system (169). Physiologically, Nrf2 is the pivotal antioxidant gene activator of the Nrf2/Keap1/ARE pathway, which is restrained at low level due to its combination to Keap 1. Nrf2 is detached from Keap 1 at the stimulation of oxidative stress, and enters the nucleus to accumulate and combine with ARE; subsequently, maintenance of balance of the oxidation/antioxidant occur *via* transcription of series of antioxidative protein genes including glutathione (GSH), NAD()H quinone dehydrogenase 1(NQO1), and heme oxygenase 1 (170). Few ncRNAs are recognized to be associated with regulation of Nrf2 and have been characterized in other diseases, including MALAT1 (169, 170). The silencing of MALAT1 showed significant reduction in cell viability and apoptosis enhancement in isoproterenol (ISO)-treated H9C2 cells. Additionally, MALAT1 acted as sponge in a functional study by decreasing ULK1 expression and repressed ISO-induced protective autophagy. The knock-down of MALAT1 showed decreased expression of miR-22-3p target gene CXCR2 *via* the function of miR-22-3p, thereby leading to augmentation of oxidized low-density lipoprotein (ox-LDL)-induced endothelial injury. A contrary report on the protective role of MALAT1 showed the increase in apoptosis and oxidative stress in human lens epithelial cells *via* p38 MAPK pathway in diabetic cataract. The expression of H19 was up-regulated in patients with atherosclerosis. The silencing of H19 enhanced cell viability, and repressed interleukin (IL)-1β, IL-6, as well as tumor necrosis factor (TNF)-α and down-regulated VCAM1, ICAM1, and E-selectin. The silencing of H19 also decreased ROS levels. H19 can increase H_2_O_2_-induced degenerative changes in intervertebral disc *via* the enhancement of cell senescence and inhibiting nucleus pulposus cell proliferation by activating Wnt/β-catenin signaling (171). Though the up-regulation of H19 has been reported in numerous tissues/cells in oxidative stress; studies have also shown the down-regulation of this lncRNA in rat models of diabetic cardiomyopathy (DCM). H19 is up-regulated in diabetic rats, thereby reducing oxidative stress, apoptosis, and inflammation, leading to the function of amended left ventricular. The down-regulation of H19 has been linked with high glucose levels. The regulation of high glucose-induced apoptosis in DCM showed H19/miR-675 as the underlying mechanism (171). As stated earlier, experimentally induced colon carcinogenesis in a rat and CRC cells showed interplay of the PO with miR-126/VAM-1 and miR-12/PI3K/AKT/mTOR, this is identified as a mechanism which in part mediate anti-proliferative and anti-inflammatory activity of PO polyphenols (134). Therefore, the mechanisms relating detoxification by glutathione and miR-12/PI3K/AKT/mTOR, can be a useful tool in the diagnosis and treatment of colorectal cancer. However, the communicating element between PO and miR-126-1 and miR-12/P13K/AKT/mTOR need to be elucidated. The participation of oxidative stress in human disorders cannot be overemphasized, and the maintenance of the balance between the elimination and accumulation of ROS is an indispensable cell function for its survival. Several transcription factors are induced by oxidative stress, including Nrf2, NF-κβ, HIF-1α, β-catenin/Wnt, AP-1, PPAR-γ, and p53. The expression of several genes is activated by these factors: and thus, influencing immune responses, growth factors, and cell cycle transition. Nrf2 regulates the transcription of glutathione peroxidase and glutathione S-transferase (GST), glutathione reductase, glutamate-cysteine catalytic subunit (GCLC), glutamate-cysteine ligase modifier subunit (GCLM) (172). Contrarily, the modification of cysteine residues on KEAP1 occur under oxidative stress, this alters and disrupt the association of KEAP1 with Nrf2, thereby stabilizing Nrf2 (39). Thus, leading to translocation of free Nrf2 to the nucleus, and Nrf2 binds to antioxidant response elements throughout the genome, activating downstream effector genes (39). Additionally, degradation of Nrf2 is regulated by β-TrCP-dependent pathway, through phosphorylation of Nrf2 by GSK3 in a PI3K-dependent manner (173). Resulting phosphorylation of Nrf2 is ubiquitinated by β-TrCP and degraded by proteasome, further effect on Nrf2 activity occurs *via* simultaneous inactivation of phosphatase and tensin homolog (PTEN; a phosphoinositide 3-phosphatase) (174). Mutations in Nrf2 or KEAP1 results in metabolic rewiring of tumors which leads to increased glutamine consumption for GSH synthesis (175, 176). Melanoma and lung cancers patients suffer from high levels of GCLC (177) as well as elevated enzymatic activity of GCLC in renal cell carcinoma patients (178). *MiR-18a* induced by Myc attenuates the expression of GCLC (179). The regulation of GCLC expression by Myc also occurs in effector T cells and this can regulate immunotherapeutic responses in cancer patients (180). Cell growth attenuation in esophageal cancer and apoptosis promotion in ovarian and breast cancers is enabled by irreversible GCL inhibitor-buthionine sulfoximine (BSO) *via* reduction in GSH production (181). There is current use of BSO in combination therapy in clinical trials, to determine patients’ response to melphalan in resistant neuroblastoma (179, 182).

This review has focused on glutathione as the detoxifying mechanism and its influence in colorectal cancer development and progression. The elucidation of the cross-talk between the oxidative response by glutathione, Nrf2, mTORC1, HIF, and the intestinal ecosystem is required to better understand the response of cells to dietary factors. ncRNAs are involved in oxidative stress response, but there is specificity in cell types in the involvement of ncRNA. It is shown in this review that there exist communicating abilities within the intestinal ecosystem, the understanding of the specificity of communication within the system during oxidative stress is not known. “The balance between Nrf2/GSH antioxidant mediated pathway and DNA repair modulates cisplatin resistance in lung cancer cell. Silva & colleagues reported that cisplatin cytotoxicity is determined by intracellular levels of glutathione and by the activity and expression of Nrf2. The analysis of gene expression in non-small cell lung cancer patients of TCGA bank revealed significant lower overall survival in patients bearing tumors with unbalanced level of Nrf2/KEAP1 and increased expression of Nrf2 target gene, thus showing the importance of glutathione and Nrf2 levels as biomarkers in cisplatin resistance in lung cancer” (172). Although the involvement of glutathione in response to pathogenic infection is known in limited bacterial infection, the understanding of the response to microbiota that facilitate CRC development is unknown. This can be a research interest that will facilitate the design of therapeutics for the prevention and treatment of colorectal cancer.

However, the understanding that the intestine is an ecosystem consisting of microbiota of varying phyla brings to mind the question on the implication of GST polymorphism on microbiota occurrence in the intestine and the implication of the influence of GST polymorphism on microbiota occurrence in CRC development and progression. Also, worth noting is the influence of GST gene polymorphism on Nrf2/mTORC1/P13K cross-talk, could this understanding be the key to resistant in treatment? Additionally, what impact does polymorphism of GST has on CRC treatment, can diet correct GST gene polymorphism, since high red meat intake in a GST gene polymorphism increases the risk of CRC. Also, is GST gene polymorphism the reason for recent increase in occurrence of CRC in young adults? other than the hereditary factors of APC?. There is limited study on inter kingdom transfer of ncRNAs and exosomes related to diets/nutrient and humans

Exosomes in plant and animal contribute to innate immunity and mediate intercellular communication. These exosomes in the intestinal ecosystem are identified by their cargo, and due to their non-degradability in low pH, can also be used as therapeutic vehicles to targets. “The loading of exosomes with cargos is not a random process but involves sorting mechanisms that favor some cargos over others. Exosomes may deliver their cargos over short distances to receptors adjacent to the exosome-secreting donor cell, or cargos may be delivered to receptor cells in distant tissues” (183).

## Conclusion

This review has indicated the influence of dietary production of free radicals in the intestinal ecosystem. The content of Glutathione is influenced by sulfur nutrition and polymorphism and the adjustment in its status can be triggered by various stresses (184). Understanding the interplay that diet has on the intestinal ecosystem, is an important factor that will determine the design of therapy and aid monitoring of prognosis. What exact player are we looking for in the possible biomarker for early detection and intervention in colorectal cancer development and progression?

The answers are in the communication machinery between cells and across kingdom. The interplay between defense against oxidative stress, immunity, and defense by the gut bacteria, fungus, and virome is required. We propose that early detection of CRC should involve exosome analysis of defense mechanisms such as glutathione and the prerequisite enzymes for glutathione synthesis. The virome, fungi, and protozoa of the gut needs to be elucidated to understand the complexity of the interplay in the gut ecosystem. Understanding of the role of the virome will give insight into targeted therapy particularly due to the current personalized medicine (patient specific therapy). Also, the understanding of the exosome assisted miRNA in dietary factors and in virome can result in early diagnosis of colorectal cancer to bring about early intervention and treatment. Although attention was only given to exosomes, however, much consideration need be given to dietary ncRNAs and microvesicles to understand the interplay of microbiota-virome adherence to the host; this will facilitate the understanding of involvement of TLRs that is modulated by virome and exosome miRNAs. Dietary bioactive components such as chemical component is the current understanding of how diets impact on human health, considering that most vegetables and fruits are eaten as uncooked meal, is transfer of dietary ncRNAs a fairy tale?

The understanding of the sequel of action will aid the diagnosis and determine prognosis. Can oral occurrence of microbiota be a diagnostic and prognostic biomarker to colorectal cancer incidence? Can we continue to rely on CEA value for the diagnosis and prognosis of colorectal cancer? Although it is known that GSH and GSH-related moieties are vital in tumor initiation (185), progression, and resistance to drug, there is less clarity to its distinct role, and the orchestration between GSH, metabolism and the microenvironment has not been elucidated

Prevention of CRC can be *via* gene profiling to determine the GST gene status of an individual and thereby prevent such an individual from CRC *via* gene therapy. Is GST polymorphism a factor to consider in hereditary CRC.

## Author Contributions

Conception and design, development of methodology, writing: BE-A. Acquisition of data, analysis and interpretation of data, review, and/or revision of the manuscript: all authors. All authors contributed to the article and approved the submitted version.

## Funding

This work was supported by ZEAB Therapeutic LTD.

## Conflict of Interest

The authors declare that this study received funding from ZEAB Therapeutic Ltd. The funder had the following involvement with the study: study design, data collection and analysis, decision to publish, and preparation of the manuscript
